# AKT, p-AKT, ERK1/2 and p-ERK1/2 in Mural Granulosa Cells Are Not Correlated to Different Ovarian Stimulation Protocols in Patients Undergoing Assisted Reproductive Treatment

**DOI:** 10.3390/life14050554

**Published:** 2024-04-25

**Authors:** Giovanni Ruvolo, Domenica Matranga, Maria Magdalena Barreca, Liana Bosco

**Affiliations:** 1 Centro di Biologia della Riproduzione, 90141 Palermo, Italy; ruvologi@hotmail.com; 2 Department of Health Promotion, Mother and Child Care, Internal Medicine and Medical Specialties, University of Palermo, 90127 Palermo, Italy; domenica.matranga@unipa.it; 3 Department of Biomedicine, Neuroscience and Advanced Diagnostics (Bi.N.D), Section of Biology and Genetics, University of Palermo, 90133 Palermo, Italy; mariamagdalena.barreca@unipa.it

**Keywords:** mural granulosa cells, apoptotic pathway, oocyte competence, quality marker

## Abstract

(1) Background: In this paper we aim to study the relationship between the expression levels of molecules involved in apoptotic/survival pathways, considered as molecular markers of oocyte competence (i.e., AKT, p-AKT, ERK1/2, and p-ERK1/2) in mural granulosa cells (MGCs) and the administration of r-FSH alone or combined with exogenous r-LH, in ovarian stimulation protocol. Moreover, we aim to evaluate oocyte competence by comparing normally cleaved embryos that were transferred in the uterus, with embryos that were arrested during in vitro culture. (2) Methods: The study included 34 normo-responder women undergoing ICSI procedures. All subjects were divided into two groups. Group A consisted of 18 women stimulated with r-FSH and used as a control group; Group B consisted of 14 women stimulated with r-FSH combined with r-LH. The MGCs were obtained from individual follicles. Immunoblot analyses were carried out to analyze the AKT, p-AKT, ERK1/2, and p-ERK1/2 levels in MGCs and to correlate them with the ovarian stimulation protocol. Furthermore, the oocyte competence was evaluated, for each follicle, according to the development of the embryo during in vitro culture and the pregnancy outcome. (3) Results: We found no significant difference in the levels of molecules in isolated MGCs between groups A and B. These results, in light of our previous research, suggest for the first time, to our knowledge, that cumulus cells and mural granulosa cells in the same follicle show different expression levels of molecules involved in the apoptotic mechanism. (4) Conclusions: Our results could clarify some controversial data in the literature where cumulative cell pools of cumulus and granulosa were analyzed, described as ovarian follicle cells, and used as markers of oocyte competence. In this paper, we found evidence that cumulus and granulosa cells need to be analyzed separately.

## 1. Introduction

Assisted Reproductive Treatment (ART) represents an effective therapy in the treatment of male and female infertility, which affects approximately 15% of reproductive-age couples [1]. In ART protocols, oocytes are recovered after ovarian stimulation using exogenous gonadotropins. During oocyte retrieval, a variety of oocytes are collected at different stages of maturity. A critical step in ART is the assessment of the oocyte and the competence of the embryo, to select the embryo(s) that shows the greatest viability for transfer. The clinical ART outcomes, in terms of ongoing pregnancy rate, have low efficiency and do not exceed 30% for each treatment cycle. Therefore, to increase the chances of success for a given cycle, many centers perform multiple embryo transfers (ETs), significantly increasing the risk of multiple gestations. Recently, the ESHRE guidelines about the number of embryos to be transferred during IVF/ICSI highlighted that the cumulative live birth rate is not inferior in elective single embryo transfer compared to double embryo transfer [2]. However, it is associated with a significant risk of multiple gestations and a higher risk of preterm birth with possible complications, such as cerebral palsy and infant death [3]. The most widely used strategy is the careful selection of embryos with higher implantation potential, based on morphology and cleavage rate (as assessed by the embryologist or using an automatic time-lapse system) [4]. Sometimes, this is coupled with a pre-implantation genetic diagnosis. Although they are successful to some extent, these methodologies are less rigorous than desired [3]. Assessing oocyte quality by morphological criteria is controversial given the low predictive value of these methods with respect to embryo survival and pregnancy outcome [5]. Therefore, developing reliable tests to assess oocyte and embryo viability, thus improving an embryo’s implantation potential, remains one of the most significant contemporary goals of reproductive medicine. Thus, it is fundamental that the scientific community explores new biomarkers that might help to improve oocyte assessment and selection, as oocyte quality appears to be one of the most limiting factors to the application of ART cycles [3,6].

It is known that all of the folliculogenesis processes depend on bi-directional interactions between germ cells and the surrounding granulosa cells (GCs) [7]. In particular, granulosa cells (GCs) support oocyte growth by controlling and modulating the steps of meiosis and the transcriptional activity of the oocyte [8]. Furthermore, GCs are responsible for the production of steroid hormones such as progesterone and estradiol [9], and of essential nutrients indispensable for oocyte development, such as modulating carbohydrate metabolism, and lipid synthesis [9,10]. Hence, oocyte quality is heavily associated with GCs’ functions. Identifying specific molecular markers related to the activity of these cells could potentially be used to predict oocyte quality [11].

Recent studies attempted to define standard protocols for oocyte competence evaluation and to predict the outcome of in vitro fertilization, based on the incidence of GCs’ and cumulus cells’ (CCs) apoptosis. The onset of apoptosis in GCs may affect their ability to interact with each other and with the oocyte [12].

GCs, somatic cells surrounding oocytes, differentiate into CCs and mural granulosa cells (MGCs) during the antral follicular phase. CCs and oocytes are in direct contact with each other, forming a cumulus–oocyte complex (COCs), where the former provides nutrition and energy to the latter. On the other hand, MGCs are located on the follicle wall and mainly perform endocrine functions. Both CCs and MGCs play a pivotal role in the development and growth of follicles [13,14] by producing and secreting hormones, as well as growth factors, into the follicular fluid [15]. Oocyte competence is directly related to the microenvironment provided by CCs and MGCs [11]. Different studies have investigated the fertility potential of single oocytes by using non-invasive approaches in ART cycles, without compromising oocytes integrity [16]. These studies have demonstrated that a low apoptosis rate, assessed by measuring the DNA fragmentation index (DFI) and by detecting surviving pathway molecules (i.e., p-AKT) in the CCs, could be considered as a molecular marker for oocyte competence [17]. Moreover, in our previous paper, we suggested that high levels of nuclear p-ERK1/2 in the cumulus cells of corresponding COCs, coupled with an increase in p-AKT concentration and a low DFI rate, may be considered as an effective marker of oocyte competence, and may also predict blastocyst formation [18]. However, the role of the MGCs is poorly understood and the differences existing between the two types of somatic cells present in the same follicle are poorly characterized [11]. Some studies address the correlation between a higher presence in the granulosa of apoptotic cells and a higher occurrence of empty follicles, a lower retrieval rate of oocytes, lower fertilization rates, and a generally poorer quality of oocytes and embryos [19,20]. Whether MGC apoptosis has a direct relationship with oocyte quality is much more difficult to ascertain. Sadraie and coworkers [21] found a significantly higher incidence of apoptotic cells in MGCs in women > 40 years old, with fewer and less mature oocytes retrieved. Corn et al. suggested that apoptosis could impair oocyte maturation and lower the chance of blastocyst formation [22]. Recent studies showed that ERK1/2 mediates the changes in follicular cell fate induced by the LH hormone during ovulation and luteinization. On the other hand, ERK can play a role both in the survival signaling pathway and the apoptosis signaling pathway.

Many growth and survival factors can activate the AKT pathway, which in turn regulates cell survival [23]. It is largely proved that AKT promotes cell survival by interfering with apoptotic cell death [24,25]. It is also known that in each stage of follicle development, AKT activation by phosphorylation is a crucial step for the proliferation and apoptosis suppression of granulosa cells [26,27]. Although it is known that AKT regulates cell survival in different cell types, such as in human granulosa cells, its role in this process is not completely understood.

Therefore, the aim of this study was to compare levels of apoptotic/survival molecules (i.e., AKT, p-AKT, ERK1/2, and p-ERK1/2) in the MGCs upon administration of exogenous r-LH combined with r-FSH or r-FSH alone, in ovarian stimulation. Also, this study assessed the possibility to use the above factors as potential molecular markers of oocyte competence, comparing normally cleaved embryos with embryos that were arrested during in vitro culture.

## 2. Materials and Methods

### 2.1. Study Design and Patients

A total of 32 women, attending ICSI treatment because of infertility at the Centre for Reproductive Biology of Palermo (CBR, Palermo, Italy), were enrolled in this study. An informed consent was signed, which allowed researchers to use MGCs recovered after ovarian stimulation. Patients were assigned a different ovarian stimulation protocol according to clinical practice. A group of 18 women was stimulated with r-FSH and used as control group (group A), and a group of 14 women was stimulated with r-FSH combined with r-LH (group B). The couples’ inclusion criteria were as follows: normo-responder status with a minimum of six oocytes collected at pick-up, age ≤ 38 years, normal follicle stimulating hormone (FSH) basal level < 12 UI/mL, and body mass index (BMI) < 28 kg/m^2^. Azoospermia or severe oligo-asthenospermia (motile sperms < 0.1 × 10^6^/mL) were considered as an exclusion criterion, as described in our previous papers [17,18]. All procedures were in accordance with the ethical standards of the 1964 Helsinki declaration and its later amendments. This research was approved by the Internal Review Board of CBR.

### 2.2. Ovarian Stimulation

The gonadotropin releasing hormone (GnRH) agonist Buserelin (Suprefact, Sanofi-Aventis, Italy; 0.2 mL/day) was administered to all patients who presented a normal basic level of FSH (<12 IU/mL) and normal body mass index (BMI = kg/m^2^ < 28) starting on the 21st day of the previous cycle. On the 8th day after Buserelin treatment, administration of 150 IU r-FSH/day (Gonal-f, Merck-Serono, Rome, Italy) began in patients of A group, while in the patients of the B group, the administration of r-LH (75 IU/day, Luveris; Merck-Serono, Rome, Italy) began from day 8 after starting r-FSH stimulation. Follicular growth was monitored every 2 days using ultrasound and serum estradiol E2 levels, from the 6th day of stimulation, modifying the dose of r-FSH as needed. A dose of 10,000 IU hCG (Ovitrelle; Merck-Serono, Rome, Italy) was administered when at least three follicles showed a diameter of ≥18 mm. Only metaphase II (MII) oocytes, showing the extrusion of the first polar body, were fertilized by ICSI. Two different media (VitroLife, OVOIL, G-MOPS, G-IVF and G-TL media, Göteborg, Sweden) were used for oocyte and embryo cultures, using microdrops of culture medium (30 μL) in a 60 mm Falcon petri dish (Corning, New York, NY, USA) covered in paraffin oil (Ovoil). Briefly, the oocytes were cultured in G-IVF drops after the pick-up. After decumulation, the oocytes were transferred into G-TL drops until ICSI, which was carried out in buffered medium (G-Mops). Subsequently, the oocytes were transferred into G-TL drops until transfer or vitrification. All cultures were carried out at 37 °C, 6% CO_2_, and 5% O_2_. Then, 8-cells stage embryos or 5–6-day-old blastocysts were transferred or vitrified (Kitazato, Shizuoka, Japan) according to clinical indication, while embryos arrested at different cleavage stages were discarded after 8 days of culture, as described in our previous papers [17,18].

### 2.3. Granulosa Cells Preparation

Granulosa cells were obtained from each single follicle containing MII oocyte. The granulosa cells were recovered from the large clusters present in the follicular fluid aspirated during the pick-up. These large clusters were washed in Ham’s F10 medium with several passages, until any residual blood cells were completely absent and centrifuged at low speed (800 rpm for 7 min), to maintain integrity. Cells were suspended in Ham’s F10 medium, without serum albumin, to avoid interferences in Western blot analyses. From each single follicle, granulosa cells were processed for Western blotting analyses, using anti-AKT, p-AKT, ERK1/2 and p-ERK1/2 for immunoreaction. Not all MGCs for all oocytes gave complete data for all molecules analyzed, due to experimental technical limitation.

### 2.4. SDS-PAGE and Western Blot

MGCs from each single follicle were washed in PBS, pelleted and lysed in a 7 M urea, 2% CHAPS, and 10 mM DTT lysis buffer with the addition of protease inhibitors cocktail tablets (Roche, Basel, Switzerland 1836170). The lysates were centrifuged at 1000 rpm for 1 min at 4 °C. Protein concentrations were quantified through Bradford method (Bio Rad, Hercules, CA, USA) using bovine serum albumin (BSA, Sigma-Aldrich, St. Louise, MI, USA) as a standard. Immunoblots were performed according to [17,18] with minor modifications. Briefly, protein extracts of MGCs were submitted to 10% sodium dodecyl sulphate-polyacrylamide gel electrophoresis (SDS-PAGE) with subsequent protein transfer onto Hybond-ECL nitrocellulose membranes (GE Healthcare, Piscataway, NJ, USA) using a Novablot semidry apparatus (Amersham Pharmacia Biotech, Piscataway, NJ, USA). The membrane was then blocked in 5% BSA solution and incubated over night with the following primary antibodies: anti-ERK1/2 (ab17942, 1:500, Abcam, Cambridge, UK), anti-p-ERK1/2 (9101, 1:750, Cell Signaling, Danvers, MA, USA), anti-AKT (9272, 1:750, Cell Signaling technology, Danvers, USA), anti-p-AKT (sc-7985-R, 1:750, Santa Cruz Biotechnology, Santa Cruz, CA, USA), and anti-actin (A5060, 1:500, Sigma) as a loading control and normalization. The membranes were incubated with appropriate secondary antibody HRP-conjugated anti-rabbit IgG antibody (W4011, Promega, Madison, WI, USA, 1:25,000). The chemiluminescent signal was revealed using a chemiluminescence solution, ImmunStar Western C Substrate Kit (Bio-Rad) and detected using the Chemidoc XRS acquisition instrument (Bio-Rad, Hercules, CA, USA). The obtained images were analyzed with Quantity One v.4.6.6 software (Bio-Rad).

**Figure 1:**
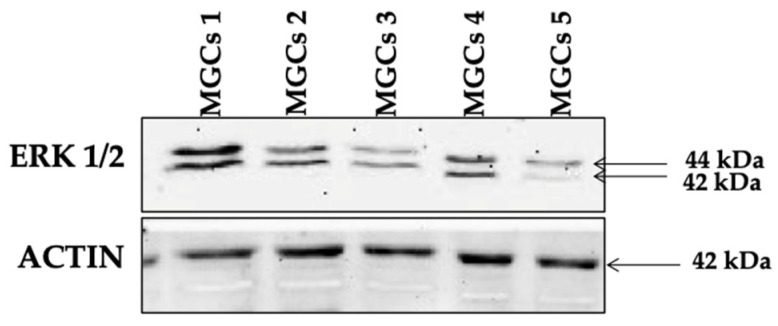
Representative Western Blot assay for ERK1/2 in MGCs isolated from each single follicle obtained from one patient.

The representative image for Western Blot assay of one of the markers studied (ERK1/2) is shown in Figure 1.

### 2.5. Statistical Analysis

In MGCs isolated from each single follicle, we analyzed the expression levels of the proteins AKT, p-AKT, ERK1/2, and p-ERK1/2, considered as apoptosis/survival markers, to verify if they correlate with oocyte competence. This was assessed by recording embryo development during in vitro culture and by observing the outcome in terms of ongoing pregnancies. Continuous variables (molecules expression levels) were expressed as median value (Me) and the interquartile range (Q1 is the 25th and Q3 is 75th percentiles), being positively skewed. Categorical values were expressed as counts or percentages (%). The statistical analysis considered the dependent information of oocytes taken from each woman. Generalized Estimating Equations (GEE) models were estimated to assess the statistical significance of the difference between molecules expression of individual MGCs of patients by stimulation protocol (group B vs. group A) and by embryo’s outcome (transferred vs. arrested). GEE models were obtained with Gamma distribution and log link, and the most appropriate model was chosen according to Quasi-likelihood under Independence Criterion (QIC). Finally, the logistic regression of pregnancy related to molecules’ expression of individual MGCs and stimulation protocol was estimated and the adjusted odds ratios (ORs) with 95% Cis were calculated. The robust Sandwich estimator of standard errors was considered to take data correlation into account.

## 3. Results

Patients enrolled in the study were 34.1 ± 4.0 years old overall, 34.0 ± 2.9 for group A and 34.2 ± 4.9 for group B (*p* = 0.853). They reported a FSH basal level of 9.7 ± 1.3 UI/mL overall, 9.6 ± 1.2 for group A and 9.8 ± 1.5 for group B (*p* = 0.3638), and a BMI equal to 22.1 ± 1.9 overall, 22.2 ± 1.7 for group A and 22.0 ± 2.0 for group B (*p* = 0.7037).

**Figure 2:**
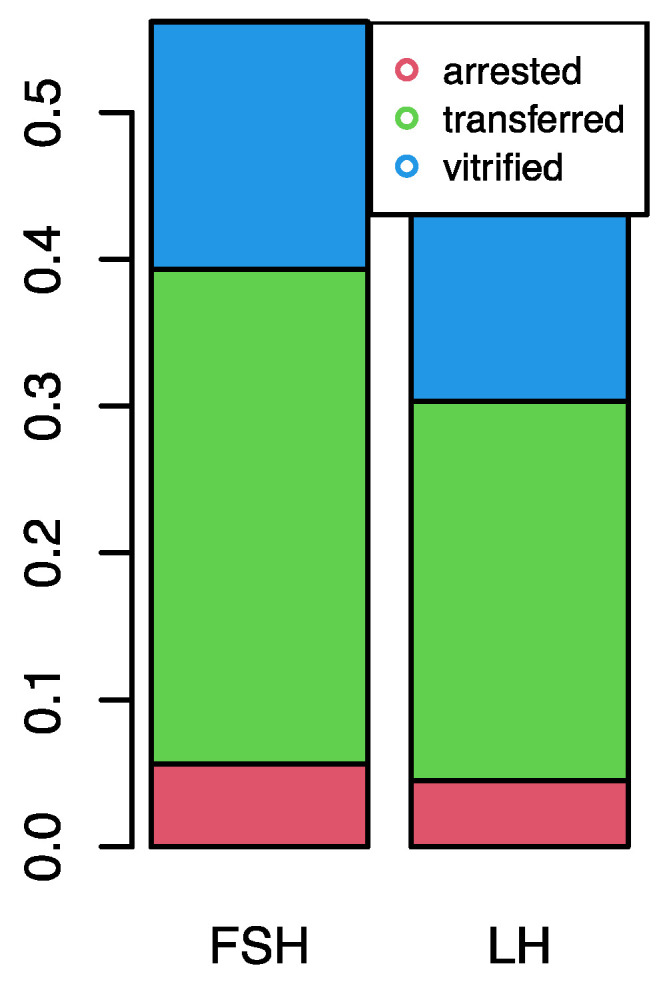
Embryo development by ovarian stimulation.

Out of 110 collected oocytes, 9 were not analyzed because of their immature condition stage as a germinal vesicle (GV). Of the remaining 101 from group A (56) and group B (45), 4 were excluded because they were at Metaphase I (MI). Of the 97 mature oocytes at MII stage, 89 (88.1%) were fertilized and the embryos developed as follows: 53 (59.6%) were transferred, 27 (30.3%) were vitrified, and 9 (10.1%) were arrested during in vitro culture. In group A, 30 embryos were transferred at 8-cells stage embryos or 5–6-day-old blastocysts and likewise in group B, where 23 embryos were transferred. Five and four embryos, in group A and B, respectively (10% in both cases), stopped development at different cleavage stages. A total of 15 embryos were vitrified in group A and 12 in group B, respectively (Figure 2).

The clinical pregnancy and implantation rate were, respectively, 33% (*n* = 6) and 36% in group A, and 43% (*n* = 6) and 42% in group B, with no statistical difference (Table 1).

In MGCs isolated from each single follicle, we analyzed the expression levels of the proteins considered as apoptosis/survival markers (AKT, p-AKT, ERK1/2, and p-ERK1/2) to verify if they correlate with oocyte competence. This was assessed by recording embryo development during in vitro culture and by observing the outcomes in terms of ongoing pregnancies.

In our experiments, none of the markers analyzed showed a significant difference in the MGCs from the follicles from group A patients compared to group B patients (Table 2).

Immunoblot analysis showed no statistically significant differences in the protein levels of MGCs isolated from follicles whose oocytes gave transferred embryos (8-cells stage embryos or 5–6-day-old blastocysts) compared with those that gave embryos that were arrested during in vitro culture (Table 3).

Finally, the AdjORs with 95% CIs for pregnancy showed no significant relation to protein levels in MGCs, adjusted for age and stimulation protocol (Table 4).

## 4. Discussion

We presented a prospective observational study to investigate the role of AKT, p-AKT, ERK1/2, and p-ERK1/2 in MGCs as apoptosis/survival molecules, by correlating their expression with the administration of exogenous r-FSH combined with r-LH in ovarian stimulation comparing with r-FSH alone, in patients undergoing ICSI treatment. Our aim was to ascertain if the presence of these molecules in MGCs could be used as a marker for oocyte competence, based on the development of embryos in vitro and on the outcome of pregnancies. Identifying competent oocytes is a critical factor in the field of assisted reproductive technology (ART), including in vitro fertilization (IVF), as it significantly influences the potential to achieve embryos with high implantation capacity. The quality and competence of an oocyte are paramount in determining the embryo’s ability to develop properly, implant in the uterus, and result in a successful pregnancy. Competent oocytes have the correct genetic material and the cellular machinery necessary for fertilization, early embryonic development, and the initiation of a healthy pregnancy. The identification of competent oocytes can dramatically enhance the efficiency of fertility treatments. By selecting the most viable oocytes for fertilization, clinicians can increase the chances of producing high-quality embryos. This selection process reduces the need for multiple IVF cycles, decreases the physical and emotional strain on patients, and can significantly lower treatment costs. In the previous decades, several technologies and methodologies have been developed to try to enhance the selection of highly competent oocytes in the field of assisted reproductive technology. Morphological assessment is the traditional method and is still widely used; it involves the microscopic examination of oocytes to assess their physical characteristics, such as size, shape, and the appearance of the cytoplasm and surrounding cumulus cells. Time-lapse imaging allows for the continuous monitoring of oocytes and developing embryos within a controlled incubation environment. Time-lapse imaging systems capture detailed growth patterns and developmental milestones, providing a wealth of data that can be used to predict oocyte and embryo viability with greater accuracy than static morphological assessment alone. Polarized light microscopy is a technique that enhances the visualization of the meiotic spindle and zona pellucida. The presence and orientation of the meiotic spindle can be an indicator of oocyte maturity and competence, as abnormalities in spindle formation are associated with chromosomal anomalies. The analysis of the metabolomic profile of the culture medium may give information on the health and viability of oocytes. Certain metabolites may be indicative of higher developmental potential. However, none of the described technologies can be considered a certain indicator of oocyte competence, to determine whether granulosa cell biomarkers may be useful in helping to select a competent oocyte to be fertilized in ART technologies.

Recently, Fan and colleagues found that in women ≥ 40 years old, there is a higher percentage of apoptosis in MGCs. They showed that MGC apoptosis is negatively correlated with the number of MII oocytes, the number of good embryos, and the number of frozen embryos [28]. However, it is known that apoptosis and reactive oxygen species production in GCs have no significant impact on fertilization and do not correlate with blastocyst development [29]. Most of these studies focused on only granulosa cells isolated from follicular fluid during oocyte retrieval. To date, the impact of the granulosa cells’ apoptosis on oocyte quality and ovarian response is still controversial. Understanding the correlation between granulosa cell apoptosis, oocyte competence/maturity, and embryo health may allow scientists and clinicians to devise better ovarian stimulation protocols for infertility treatments [30]. It is conceivable that MGCs biomarkers may be utilized for oocyte selection, prior to oocyte insemination. Among the different molecules known to support survival pathways by inhibiting apoptotic processes, a fundamental role for p-ERK1/2 and p-AKT factors was confirmed.

Our previous results [17] proved the inverse correlation between DFI and p-AKT accumulation in cumulus cells by suggesting them as molecular markers of oocyte competence, with prognostic implications concerning blastocyst formation. In addition to this, previous results by Ruvolo et al. [18] extended the previous observations to co-localization analysis of p-AKT and p-ERK1/2 in cumulus cells from single COCs, correlating the results obtained with the DFI and evolution of embryo development [22].

Here, we showed that there are no statistically significant differences in the protein levels of AKT, p-AKT, ERK1/2, and p-ERK1/2 in MGCs isolated from oocytes that have generated transferred embryos (at 8-cells stage embryos or 5–6-day-old blastocysts) compared to embryos who were arrested during in vitro culture. In the same way, we did not find differences in MGCs collected from the follicles derived from r-FSH ovarian stimulation compared to r-FSH + r-LH. Furthermore, there was no observed difference between protein levels and pregnancy outcomes. These results could suggest that the MGCs, at an early stage, exert an endocrine function to support the growth of the follicle and, around the ovulation time, follow a specific death pathway. This process is not influenced by the different protocol of ovarian stimulation, in contrast with cumulus cells, and is not correlated to oocyte competence, embryo quality, or clinical outcome. The expression of the anti-apoptotic molecules AKT, p-Akt, ERK1/2, and p-ERK1/2 in MGCs cannot be considered as marker of oocyte quality. These findings are different to other studies which demonstrated that MGCs DFI and specific gene expression have a relationship with oocyte competence [31,32,33]. Our current and past results suggest, for the first time, to our knowledge, that the cumulus cells and mural granulosa cells in the same follicle show different expression levels of the molecules involved in the apoptotic pathway and therefore, they cannot be considered as molecular markers to assess the competence of oocytes. Our study seems to demonstrate a completely different behavior between the cumulus cells and the granulosa cells of the same follicle. The cumulus cells seem to be more involved with the processes of maturation and acquisition of the competence of the oocyte, probably because they have a stronger connection with each other through the gap junctions. Cumulus cells appear more sensitive to the action of the supraphysiological doses of the gonadotropins during the ovarian stimulation protocol in the ART cycle, and to the different protocols used, as demonstrated by our previous studies [17,18,30].

Ultimately, in our opinion, the cumulus cells, through their physical connection with the oocyte through the gap junctions, share a destiny—towards cell death by apoptosis or towards survival pathways, with a precise molecular communication that mainly uses AKT/p-AKT, ERK1/2, and p-ERK1/2.

Conversely, the granulosa cells, which have no physical connection with the oocyte, communicate with the gamete through paracrine signals, mainly cGMP and EGF [34], which are used, after the LH surge, as mediators of meiotic recovery. After this function, the granulosa cells activate, unlike the cumulus cells, the cell death pathway by apoptosis regardless of the ovarian stimulation therapies used. The cumulus cells, on the other hand, can continue to survive, specifically in the dominant follicle in the physiological cycle, or in the follicles of highly competent oocytes in ART cycles, to accompany the cell in the fertilization processes, in which the same cumulus cells play an indispensable function for the capacitation of spermatozoa.

Compared to other published studies where cumulative cell pools of cumulus and granulosa are analyzed as ovarian follicle cells, the value of our study is in analyzing granulosa cells from each follicle, as separated from cumulus cells, to evaluate their possible use as markers of oocyte competence. However, our study has technical experimental limitations due to the small number of cells obtained from each follicle.

## 5. Conclusions

Our data seem to confirm that the granulosa cells are mainly destined to follow a death pathway, differently to the cumulus cells which could follow the death or survival pathway, according to external molecular signals. This different pattern suggests the specific role of the cumulus cells in determining the oocyte competence, probably one that determines the selection of the dominant follicle and the elimination by atresia of the other follicles activated at the follicular phase of the natural cycle. These considerations could clarify some controversial data in the literature, where cumulative cell pools of cumulus and granulosa are analyzed, described as ovarian follicle cells, and used as markers of oocyte competence.

## Figures and Tables

**Table 1:** Effect of two different stimulation protocols on oocytes developmental competence.

Group	A	B	*p* Value
Patient, *n*	18	14	*p* = 0.480
Total oocytes, *n*	56	45	*p* = 0.562
MII oocytes, *n*	54	43	*p* = 0.917
Nr. fertilized oocytes, *n* (%)	50 (93)	39 (91)	*p* = 0.830
Nr. arrested embryos, *n* (%)	5 (10)	4 (10)	*p* = 0.982
Nr. transferred embryos, *n* (%)	30 (60)	23 (59)	*p* = 0.956
Nr. vitrified embryos, *n* (%)	15 (30)	12 (31)	*p* = 0.965
Nr. clinical pregnancies, *n* (%)	6 (33)	6 (43)	*p* = 0.581
Implantation rate, %	36	42	*p* = 0.722

**Table 2:** Expression of molecular markers in cells from groups A and B.

	Group A r-FSH (*n* = 18) (MII Oocytes = 54)	Group B r-FSH + r-LH (*n* = 14) (MII Oocytes = 43)	*p*-Value ^§^
	Median (Q_1_–Q_3_)	Median (Q_1_–Q_3_)	
AKT	4.08 (1.55–10.20)	2.51 (1.49–9.61)	0.683
p-AKT	5.22 (3.13–11.67)	3.96 (1.73–11.92)	0.410
ERK 1/2	3.92 (2.02–10.32)	3.05 (1.82–10.59)	0.750
p-ERK1/2	5.14 (2.30–11.16)	6.59 (2.96–12.45)	0.743

^§^ *p*-value from the GEE Model. *p* < 0.05 for statistical significance.

**Table 3:** Protein levels in MGCs, from follicles whose oocytes gave transferred or arrested embryos.

	Arrested (*n* =9) Median (Q_1_–Q_3_)	Transferred (*n* = 53) Median (Q_1_–Q_3_)	*p*-Value ^§^
AKT	5.22 (3.55–11.25)	3.45 (1.49–8.76)	0.350
p-AKT	7.54 (3.73–12.24)	4.63 (1.98–11.05)	0.876
ERK1/2	4.75 (2.67–7.09)	3.06 (1.93–10.40)	0.369
p-ERK1/2	4.02 (0.73–5.85)	4.93 (2.83–10.37)	0.424

^§^ *p*-value from the GEE Model. *p* < 0.05 for statistical significance.

**Table 4:** Logistic regression of pregnancy related to protein levels in granulosa cells, adjusted for stimulation protocol: adjORs and 95% Cis.

	AdjOR (95% CI)	*p*-Value
AKT	1.03 (0.95–1.12)	0.429
p-AKT	0.98 (0.96–1.01)	0.249
ERK1/2	0.98 (0.94–1.02)	0.290
p-ERK1/2	0.98 (0.95–1.01)	0.150

## Data Availability

The datasets used and/or analyzed during the current study are available from the corresponding author on reasonable request.
